# Genetic Variation Shapes Protein Networks Mainly through Non-transcriptional Mechanisms

**DOI:** 10.1371/journal.pbio.1001144

**Published:** 2011-09-06

**Authors:** Eric J. Foss, Dragan Radulovic, Scott A. Shaffer, David R. Goodlett, Leonid Kruglyak, Antonio Bedalov

**Affiliations:** 1 Clinical Research Division, Fred Hutchinson Cancer Research Center and Department of Medicine, University of Washington School of Medicine, Seattle, Washington, United States of America; 2 Department of Mathematical Sciences, Florida Atlantic University, Boca Raton, Florida, United States of America; 3 University of Massachusetts Medical School, Worcester, Massachusetts, United States of America; 4 Department of Medicinal Chemistry, University of Washington, Seattle, Washington, United States of America; 5 Lewis-Sigler Institute for Integrative Genomics and Department of Ecology and Evolutionary Biology, Princeton University, Princeton, New Jersey, United States of America; University of California Berkeley, United States of America

## Abstract

Networks of co-regulated transcripts in genetically diverse populations have been studied extensively, but little is known about the degree to which these networks cause similar co-variation at the protein level. We quantified 354 proteins in a genetically diverse population of yeast segregants, which allowed for the first time construction of a coherent protein co-variation matrix. We identified tightly co-regulated groups of 36 and 93 proteins that were made up predominantly of genes involved in ribosome biogenesis and amino acid metabolism, respectively. Even though the ribosomal genes were tightly co-regulated at both the protein and transcript levels, genetic regulation of proteins was entirely distinct from that of transcripts, and almost no genes in this network showed a significant correlation between protein and transcript levels. This result calls into question the widely held belief that in yeast, as opposed to higher eukaryotes, ribosomal protein levels are regulated primarily by regulating transcript levels. Furthermore, although genetic regulation of the amino acid network was more similar for proteins and transcripts, regression analysis demonstrated that even here, proteins vary predominantly as a result of non-transcriptional variation. We also found that cis regulation, which is common in the transcriptome, is rare at the level of the proteome. We conclude that most inter-individual variation in levels of these particular high abundance proteins in this genetically diverse population is not caused by variation of their underlying transcripts.

## Abstract 2

Variation in the levels of co-regulated proteins that function within networks in an outbred yeast population is not driven by variation in the corresponding transcripts.

## Author Summary

The level of protein produced by each gene corresponds approximately to the level of mRNA transcript produced by that gene: so high-abundance proteins, like those involved in protein synthesis, are represented by high-abundance transcripts, whereas low-abundance proteins, like those involved in signaling pathways, are represented by low-abundance transcripts. Furthermore, genetic variation can cause variation in transcript levels for the same gene between different individuals. These two observations have led to the assumption that inter-individual variation in transcript levels for any particular gene causes corresponding variation in protein levels. However, this need not be the case, because protein levels could be controlled not only by regulating transcript levels but also by regulating protein translation and stability. Because inter-individual variation in the levels of the transcript for any particular gene is typically less than 3-fold, rather than orders of magnitude, it is possible that the predominant cause of inter-individual variation in levels of any particular protein is transcription-independent regulation of protein levels. Here, we look in a genetically diverse population of 95 yeast strains at the genetic variation that leads in turn to variation in levels of 354 proteins that function within co-regulated networks. We find that the between-strain variation predominantly reflects transcription-independent mechanisms. If this result is typical of the proteome as a whole, it suggests that protein levels in genetically diverse populations cannot be accurately inferred from levels of their underlying transcripts.

## Introduction

Genetic variation leads to networks of co-regulated transcripts. The implications of these network structures have been discussed extensively, generally with the assumption that such transcriptional networks give rise to corresponding protein networks [1]–[6]. However, due to limitations in technology, these hypothesized protein networks have not been examined directly, and thus it is not known whether they are driven by underlying transcriptional networks. By measuring protein and transcript levels for 354 genes in a genetically diverse population of yeast segregants, we are now able to address the question “Are protein networks formed primarily on the basis of regulation of their underlying transcripts?” (We use the term “protein networks” to refer to groups of proteins that are co-regulated and not groups of proteins that interact physically or genetically.) Before describing our results, three points are important to consider: First, the magnitude of individual to individual variation in transcript levels for a single gene is generally far less than the magnitude of gene to gene variation in transcript levels within a single individual [7]. Second, the demonstration in multiple studies that the correlation between transcript and protein levels for different genes within a single individual is high does not imply that differences in abundance of the same transcripts between different individuals must cause corresponding variation in protein abundance [8],[9]. And third, a correlation between transcript and protein networks does not prove a causal relationship between the two.

**Figure 1:**
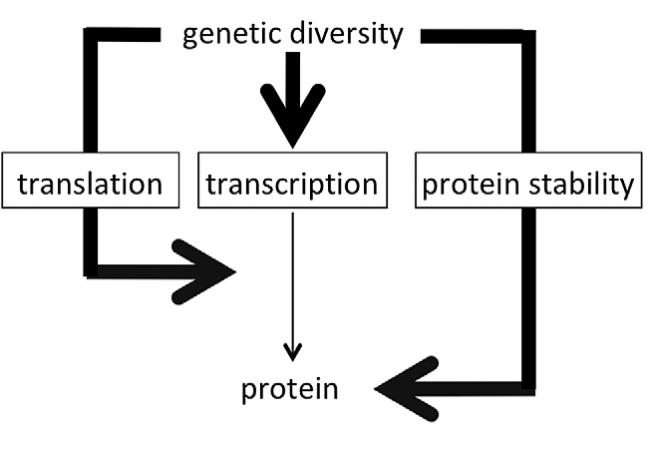
Three possibilities for regulating protein levels. Protein levels can be regulated through control of transcript levels, translation, and protein stability. The weight of the arrows here indicates the size of the effect. In this example in which protein levels are controlled primarily non-transcriptionally, there will nonetheless be a correlation between protein and transcript levels.

Protein levels cannot necessarily be inferred from transcript levels because protein levels can be controlled not only by regulating transcripts but also by regulating other steps in protein metabolism, such as translation and protein stability. Thus the degree to which protein levels can be inferred from transcript levels depends on the degree to which the former mode of regulation overwhelms the latter two (Figure 1). In experimental situations when a gene is placed under a strong promoter like the CMV promoter, a transcript can be elevated 1,000-fold and this generally leads to a striking increase in protein. However, in genetically diverse populations, transcript levels generally do not vary 1,000-fold between individuals; for example, in the population of yeast described in this report, a typical transcript varies just 2.7-fold across 95 individuals. Such modest variation in transcript levels may be buffered such that it causes no variation in protein levels, or regulation of translation and/or protein stability may obscure effects of minor transcriptional variation. Under such circumstances, transcript levels should not be expected to reflect protein levels, and whether such circumstances are the norm or the exception for typical levels of inter-individual variation is not known.

Several reports have demonstrated that, when comparing different genes whose transcript levels vary over orders of magnitude, high abundance proteins are associated with high abundance transcripts and vice versa [8]–[10]. There are two important differences between this issue and the issue we address here: First, the magnitude of variation in transcript levels is vastly different in the two situations. Just as 1,000-fold overexpression of a transcript through experimental manipulation is virtually guaranteed to increase the level of the corresponding protein, transcript levels that differ by orders of magnitude between genes are virtually guaranteed to manifest themselves in differences in the corresponding proteins. For example, a 2007 study by Lu et al. of 346 genes in yeast demonstrated a high correlation (R = 0.85) between transcript and protein levels, and thus concluded that “… >70% of yeast gene expression regulation [occurs] through mRNA-directed mechanisms” [8]. However, if one calculates correlation coefficients with this same data set using a sliding window within which transcript levels vary on average just 3.5-fold, the average correlation drops from 0.85 to 0.36 with almost half of the bins showing correlation coefficients that could easily have been achieved by chance (Figure S1, Text S1). Thus, the striking correlation between transcripts and proteins all but disappears when analysis is limited to a range of transcript variation similar to that occurring between individuals. Second, as we will discuss further below, studies like that of Lu et al. involve protein and transcript measurements from a single individual under a single experimental condition, making it impossible to measure gene-specific correlation coefficients (Figure S2). The point here is not to call into question the solid conclusions of these past reports, but instead to point out that our work addresses a different issue.

In assessing the importance of transcriptional regulation in determining protein levels, it is important to distinguish correlation from causality. Biological pathways that sense physiological conditions can trigger responses that include changes in transcription, translation, and protein stability, often with the same group of genes targeted by more than one of these regulatory mechanisms [11]. For example, the TOR pathway, a highly conserved pathway named for the signaling kinase “Target of Rapamycin,” responds to changes in nutritional conditions by increasing both transcription and translation of a group of target genes [12],[13]. Thus transcript and protein levels of these genes will be correlated, but if translation has a much larger effect on protein levels than does transcription, this correlation need not reflect causality. More generally, any time a cellular response pathway has both a transcriptional and a post-transcriptional branch, the target genes are expected to show a correlation between transcript and protein levels, but it is only in those cases where the former regulatory mechanism is the dominant one in affecting protein levels that this correlation reflects a predominantly causal relationship between transcript and protein levels (Figure 1).

## Results

### Protein Quantitation

We have previously reported a mass spectrometry-based method for protein quantitation that relies on mathematical alignment of ion signals in mass spectra (MS1) from multiple samples [14],[15]. This algorithm rounded mass to charge measurements to integer “Dalton” values, which has the advantage of making the data sets much smaller than they would be if one made full use of the high mass accuracy of modern mass spectrometers (like that on which the data were collected) and thus avoids computational difficulties that arise with large data sets. However, such rounding sacrifices accuracy to the extent that our previous quantitation, while sufficient for obtaining a broad view of the genetic architecture of protein expression, was insufficient in terms of both accuracy and coverage to rigorously address the causal relationship between variation in transcript levels and variation in the corresponding proteins. To overcome the limitations of our previous algorithm, we used a modification of an accelerated random search [16] to solve computational challenges and developed a new protein quantitation algorithm that exploits the high mass accuracy and resolution of modern mass spectrometers (Text S1). We then used this algorithm to reanalyze our previously reported [14] mass spectrometric data, aligning 380 data sets: two technical replicates of two biological replicates for each of 95 progeny strains derived from a cross between a wild type and a laboratory strain of yeast [17]. These two strains differ at approximately 0.5% of their base pairs [18], and this cross has been studied extensively [19]–[21]. Restricting ourselves to peptides that were identified with high confidence (Text S1) and that corresponded uniquely to one protein, we quantified 354 proteins (Table S1). This is more than twice the number of unique peptides (164) we were able to quantify in our previous report [14].

If one is to assess the effect of transcriptional variation on the proteome, it is necessary to focus on transcripts that show significant individual-to-individual variation. With measurements for only 354 proteins, constituting less than 6% of the proteome, we were concerned that the corresponding transcripts might not show significant individual-to-individual variation; thus we looked at variance of the transcripts in question (transcript data previously reported [17]). The 354 genes for which we had protein measurements were all among the most highly variant ∼10% (522/6,215) of all transcripts. Thus this subset of proteins is not merely sufficiently variable for our study; it comprises almost 70% (354/522) of the ideal genes on which to focus for our purposes. We speculate that this fortuitous result reflects the fact that we are best able to measure high abundance proteins, and thus our data set is enriched for accurately measured high abundance transcripts as well. (Levels of highly abundant proteins tend to be less variable than low abundance proteins [22]; therefore it is unlikely that the high variance of this set of proteins is a reflection of their abundance.) We note, however, that this is a special set of proteins in that they are mostly high abundance proteins involved directly or indirectly with protein synthesis and thus they may not be representative of the proteome as a whole.

### Construction of Genetic Networks

We next constructed a connectivity matrix between proteins on the basis of Pearson's correlation coefficient. For each pair of proteins, we used permutation testing to determine a false positive rate (FPR) cutoff, accepting only connections that were below a 1% FPR cutoff. Out of 62,481 possible protein-protein connections, we observed 7,058 connections, 91% of which were deemed genuine because we expect only 625 connections by chance. The numbers of connections for individual proteins ranged from 1 to 100, with an average of 40 and a median of 37. For transcripts, we observed 15,989 connections out of 62,481 possible. For individual genes, the transcript numbers ranged from 4 to 176, with an average of 90 and a median of 76. Among the 50 most highly connected proteins, there was a 1.9-fold enrichment for genes involved in amino acid biosynthesis, and among the 50 most highly connected transcripts, there was a 2.7-fold enrichment of genes involved in ribosomal functions, but enrichment for other functions was not obvious. Remarkably, the most highly connected genes for proteins and transcripts look entirely unrelated. For example, among the 34 most highly connected genes in the two groups, there are only two genes present in both groups (RPS7A and TEF4). A global comparison suggests that connectivity of genes at the transcriptional level is unrelated to their connectivity at the protein level (Figure S3).

**Figure 2:**
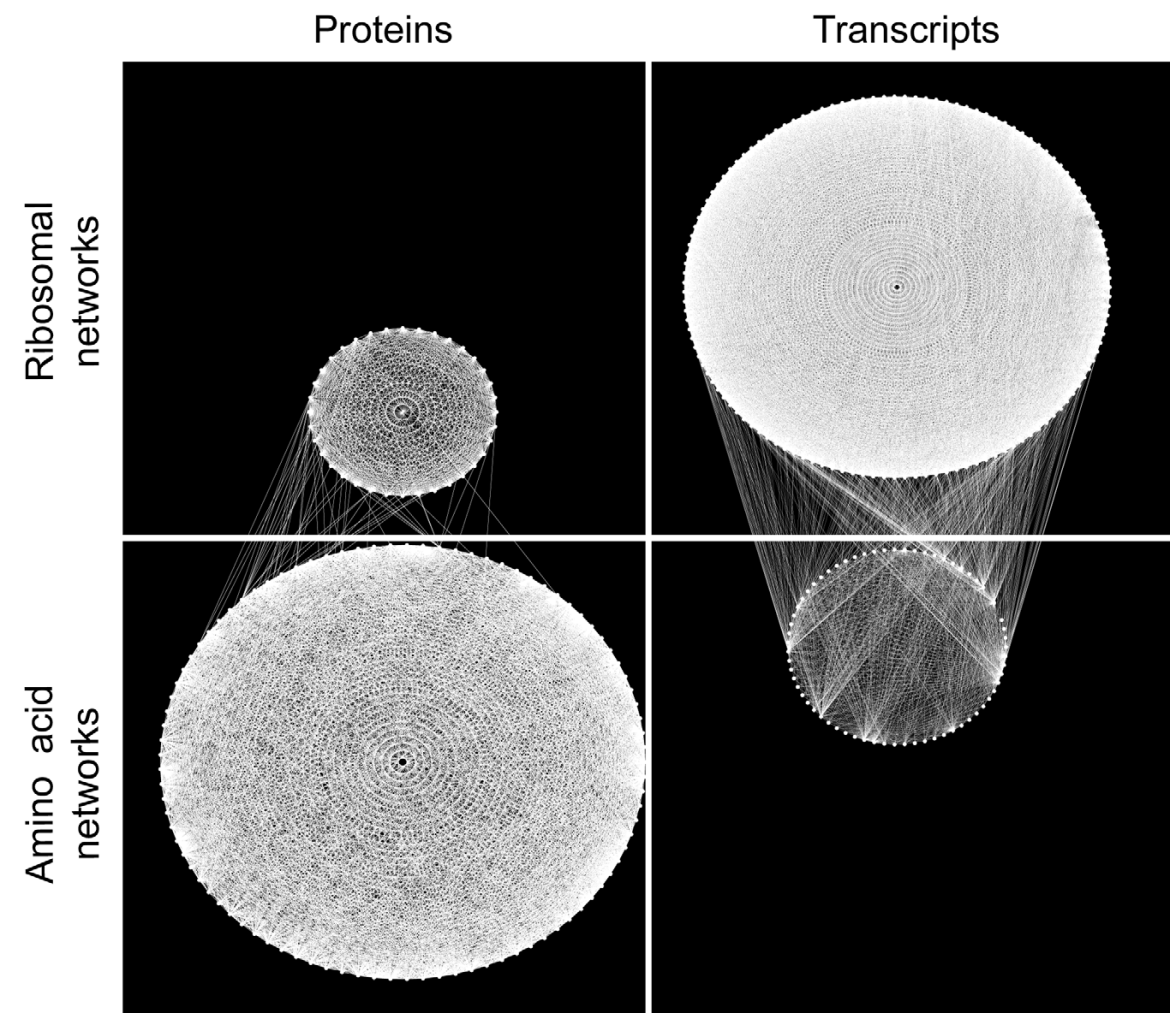
Networks of protein-protein and transcript-transcript co-regulation. Communities are defined on the basis of *k*-cliques, complete (and in our case undirected) subgraphs of size *k*, and are comprised of the union of all *k*-cliques that can be reached from each other through a series of adjacent *k*-cliques (where adjacency means sharing *k*−1 nodes). The most stringently defined (highest *k* value) protein community was a 35-clique community notably enriched for genes involved in amino acid metabolism. Lowering the *k* threshold to 19 simply expanded this community whereas at *k* = 18, there appeared five closely related communities (which we will henceforth refer to as a single community) that were enriched for ribosomal proteins. An analogous approach with transcripts yielded communities of 67 and 127 genes involved in amino acid metabolism and ribosome biogenesis, respectively. No gene was in both protein communities and just two genes were in both transcript communities. Communities of co-regulated proteins are on the left in blue and communities of co-regulated transcripts are on the right in green. In both data sets, the ribosomal community is above the amino acid community. Connections are plotted only for the 160 most highly connected genes within each data set, regardless of those genes' membership in any community. The two genes that are present in both transcript communities, VAS1 and YHR020W, are arbitrarily plotted in the ribosomal community and connections involving these genes are not plotted.

In order to identify networks of co-regulated genes, we turned to a widely used “community”-based approach [23]. Cliques are groups in which each member is connected to every other member, and “communities” are simply groups of highly overlapping cliques (precise definition in legend to Figure 2); thus in our case, communities are groups of proteins or transcripts that show a high degree of co-variation. In both the protein and transcript data sets, we identified two large communities, one enriched for genes involved in amino acid metabolism and the other for genes involved in ribosome biogenesis. The amino acid and ribosomal communities in the protein data set consisted of 93 and 36 genes, respectively, and these two communities in the transcript data set consisted of 67 and 127 genes, respectively (Figure 2; Table S2). Even though the genes within each community showed a high degree of co-regulation, the two communities within each data set showed very little connection. For example, only 1.6% (54 out of 3,348 with 378 expected by chance) of possible intercommunity protein pairs were connected; thus we have two networks of highly connected proteins that vary largely independently of one another (Figure 2). (Below, unless specified otherwise, if we refer to a ribosomal network or community, we mean the *protein* ribosomal community and the same is true for references to an amino acid network or community.)

With two large networks of functionally related proteins, we were in a position to address our main question, namely “to what extent are protein networks shaped by regulation of their underlying transcripts?” If protein networks are shaped primarily by variation in their underlying transcripts, we would expect (1) high correlations between proteins and transcripts, (2) similar genetic regulation of proteins and transcripts, and (3) that normalization of protein levels according to variation in transcript levels should abolish linkage to genetic regulatory loci. Using these criteria, we found that both protein networks were formed primarily through non-transcriptional mechanisms.

### Protein-Transcript Correlations

We calculated correlation coefficients between protein and transcript levels for all 354 genes and then assigned each a binary value of “significant” or “not significant” on the basis of 1,000 permutations done separately for each gene. Only 4 out of 36 genes in the ribosomal network showed a significant correlation (*p*<0.05); thus clearly for the vast majority of these genes, protein levels vary without regard to the levels of their corresponding transcripts. The genes in the amino acid network showed a higher fraction of significant correlations, but even here less than half of the genes (41 out of 93) showed a significant correlation at the same 5% cutoff. These results demonstrate an important non-transcriptional component to regulation of both networks and suggest that the ribosomal network is either largely unaffected by transcriptional variation, within the range of transcriptional variation observed here, or that transcriptional regulation of protein abundance is obscured by regulation of translation and/or protein stability.

The variable correlation between transcripts and proteins for these two networks raises the broader question of how transcripts and proteins are correlated in general. As noted above, several studies in yeast including one from our laboratory have reported a wide range (0.34–0.98) of correlations between protein and transcript levels [8],[9],[14]. However, two features of the current study are critically different from the previous reports. First, this study examines gene-specific correlations. Most previously reported correlation coefficients for protein and transcript abundance for yeast were derived from single measurements of protein and transcript levels for many genes in a single strain, and these individual measurements for different genes were combined to derive an average correlation coefficient. The correlation coefficients we report here, in contrast, are derived from 95 measurements of protein-transcript pairs for each of 354 different genes (Figure S2). This is important because there are dramatic differences in the degree to which different genes are regulated at the transcript level versus the protein level. Second, while our previous study reported gene-specific correlation coefficients [14], we did not emphasize these results because our marginal ability to map protein regulators raised the possibility that these low correlation coefficients reflected inaccuracies in our protein measurements (see below for mapping results). (The ability to map significant numbers of regulators can be used as a metric to assess accuracy of measurements when FDRs are empirically determined through permutation testing [24].)

**Figure 3:**
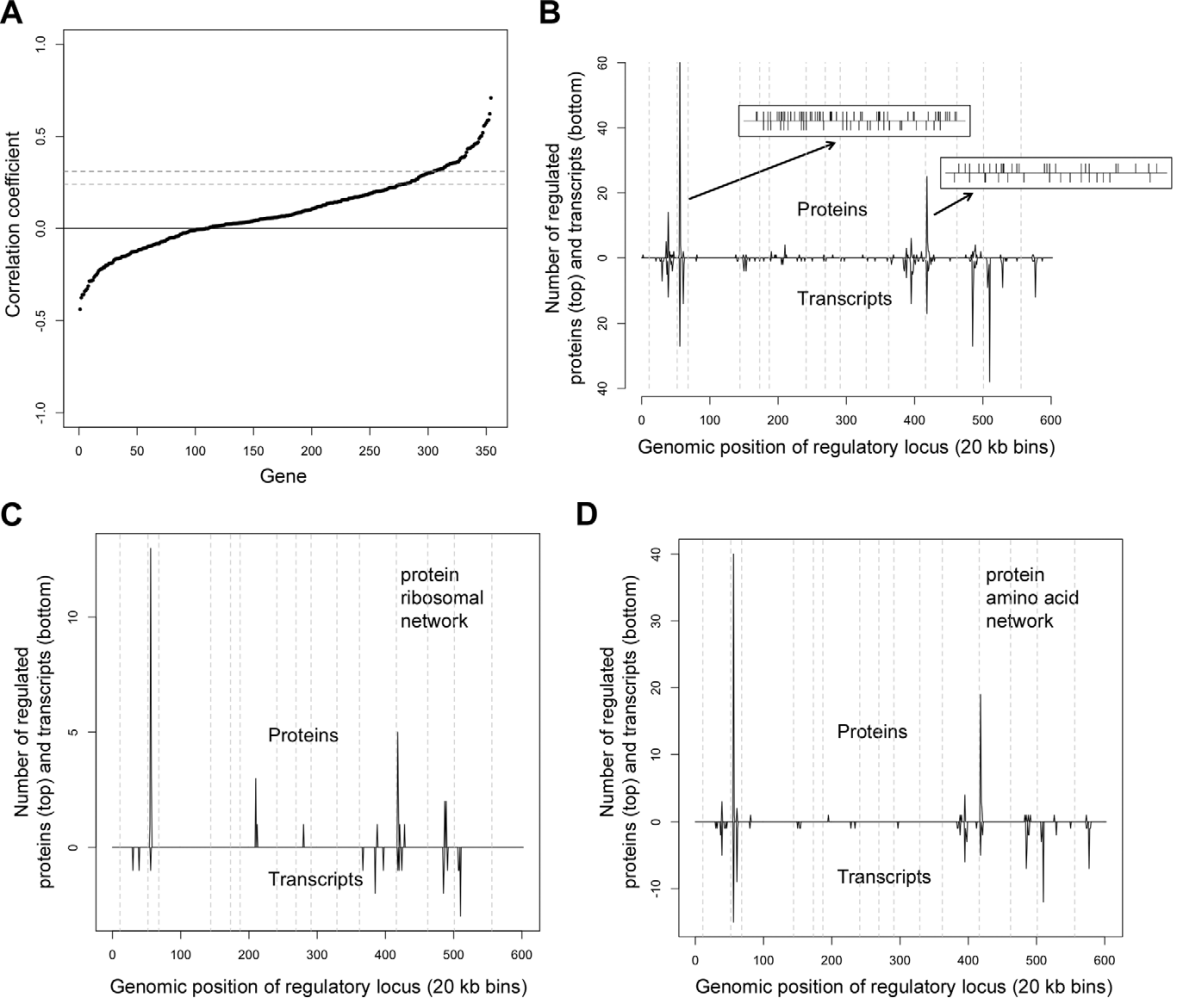
Genetic regulation of protein and transcript levels. (A) Correlation coefficients between protein and transcript levels are plotted in ascending order. The dashed lines at 0.24 and 0.31 indicate the average cutoffs for significance at *p*<0.05 and *p*<0.01, respectively, based on 1,000 permutations done separately for each gene. A very small number of genes show significant negative correlations. These may reflect protein-destabilizing polymorphisms for which the cell tries to compensate by increasing transcription, though we note that none of these loci are on the same chromosome as the regulated gene and thus any destabilization would have to act in trans. (B) Distribution of genetic regulators of proteins (top) and transcripts (bottom). The genome was divided into 20 kb bins arranged from the beginning of chromosome 1 on the left to the end of chromosome 16 on the right. The number of linkages in each bin is plotted for proteins on the top and transcripts on the bottom. Dashed vertical lines indicate the borders between chromosomes. The two insets show the locations of genes regulated by the hotspot on chromosome 3 (upper left insert) and those regulated by the hotspot on chromosome 13 (lower right insert) with proteins on the top and transcripts on the bottom. (The hotspot on chromosome 3 is likely caused by a deletion of LEU2 combined with a tightly linked polymorphism in ILV6 [43] and the hotspot on chromosome 13 is likely caused by a polymorphism in BUL2 [38]. The horizontal line in the insets represents the genomic location, going from the beginning of chromosome 1 on the left to the end of chromosome 16 on the right. (C) Locations of genetic regulators specifically for those genes in the protein ribosomal network are plotted as in (B). (D) Locations of genetic regulators specifically for those genes in the protein amino acid network are plotted as in (B).

We found that proteins and transcripts were well correlated for only 27% of genes (94/354 at 5% significance; Table S3), with most genes showing little or no correlation. Even if we limit ourselves to proteins for which we mapped regulators (*p*<0.05; see below for mapping results) and thus have high confidence in our protein measurements, only 37% of genes (46/125) show significant correlations between proteins and transcripts. Furthermore, we could find no relationship between these correlation coefficients and the corresponding genes' transcript or protein half life [25],[26]. Plotting the data in terms of variance explained for transcripts and proteins similarly failed to reveal trends (unpublished data). We conclude that for most genes, inter-individual variation in protein levels does not reflect variation in underlying transcripts (Figure 3A).

### Genetic Regulation of Networks

To compare the genetic regulation of the two protein networks, we began by mapping loci that affect transcript and/or protein levels. (We note that heritability for proteins, like that for transcripts, was high: averages for proteins and transcripts were 0.70 and 0.71, respectively, and medians for proteins and transcripts were 0.71 and 0.74, respectively; calculation described in Materials and Methods.) All strains have been genotyped for 2,955 genetic markers, 1,969 of which exhibited unique segregation patterns among the 95 segregant strains. We looked for linkage between inheritance of these 1,969 markers and the 354 transcript and protein levels using *t* tests and determined FDR cutoffs for each gene on the basis of 100 permutations. At a 5% FDR, we mapped 49 and 97 loci that control the level of a total of 125 and 200 proteins and transcripts, respectively. At a 1% FDR, we mapped 30 and 74 loci that control levels of 89 and 170 proteins and transcripts, respectively (Table S4). Because proteins and transcripts can map to more than one locus, the total number of linkages at a 5% FDR was 179 for proteins and 342 for transcripts, and at a 1% FDR these numbers were 115 and 253, respectively. These results provide an objective metric for the extent to which our current algorithm (i.e., the one used in this report) has improved our accuracy: At a 5% FDR, our previous algorithm [14] allowed us to map 24 regulators that regulate levels of 18 proteins, whereas the current algorithm allowed us to map 179 regulators that regulate levels of 125 proteins. (We use the term “regulator” to denote a locus that influences transcript and/or protein levels.) With comparable measurements for proteins and transcripts, we are now able to address questions about the relationship between the two data sets that we could not address in our previous publication [14]. Consistent with our previous results, we found that (1) both proteins and transcripts show hot spots of regulation (single loci that control multiple genes), (2) these hot spots are largely but not completely overlapping, and (3) the genes regulated by a single hot spot show low overlap at the protein and transcript levels, highlighting the difference between genetic regulation of the proteome and transcriptome (Figure 3B).

The locations of the genetic regulators that control proteins and transcripts within the ribosomal network bore essentially no resemblance to each other; indeed, given the overall distribution of regulatory loci for proteins and transcripts, loci that regulated both proteins and transcripts within this network appeared much less frequently than is expected by chance (*p*<0.0001 based on 10,000 permutations; Figure 3C). These results call into question the widely held belief that in yeast, in contrast to vertebrates, ribosomal protein levels are controlled primarily by regulation of their transcripts [27]. (We note that genetic regulation of the ribosomal transcripts is complex, i.e. a large number of loci, each with relatively small effect.) The locations of genetic regulators of proteins and transcripts were more similar for genes in the amino acid network: Approximately a quarter of the time (20 out of 84 linkages, corresponding to 17 different regulated genes), protein linkages (5% FDR) to genes in the amino acid network showed regulation of the corresponding transcript by the same locus. Below we ask whether, at least for these 20 linkages, the mechanism by which the loci regulate the proteins is regulation of their underlying transcripts.

### Normalization to Transcript Levels to Test Causality

**Figure 4:**
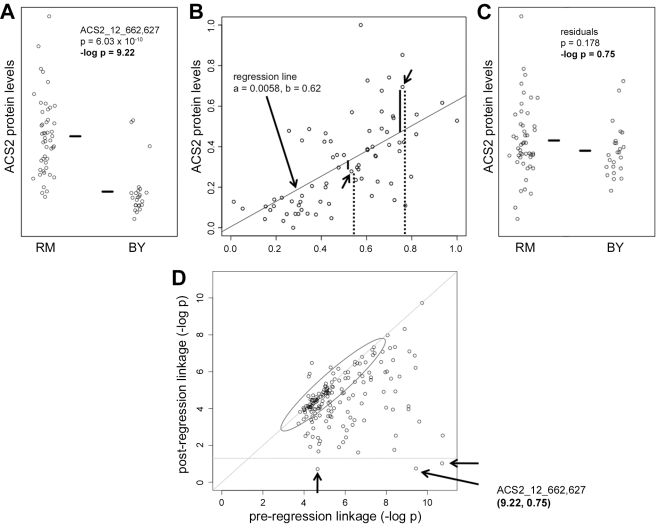
Regression analysis to test for causality. Panels A through C show an example of how the coordinates for a single point (representing the linkage between a locus at base pair 662,627 on chromosome 12 and the level of protein ACS2) in panel D were obtained. (A) The levels of protein ACS2 are higher in segregants that inherited SNP 12_662,627 from the RM (vineyard) compared to segregants that inherited this SNP from the BY (laboratory) parent. The *p* value for linkage is 6.03×10^−10^, and the negative log of the *p* value is 9.22. (B) Each segregant is plotted according to levels of the ACS2 transcript on the *x*-axis and the ACS2 protein on the *y*-axis and a regression line was calculated (slope = 0.62, y intercept = 0.0058). The regression line was used to calculate the residuals for protein levels after protein levels are regressed on transcript levels. The original values for two points (indicated by arrows) are shown as dashed lines and the corresponding residuals are shown as solid lines. (C) Same as panel A except rather than plotting the original protein levels (dashed lines in B), the residual protein levels after proteins have been regressed on transcripts (solid lines in B) are plotted. Unlike the original ACS2 protein levels (plotted in A), regressed protein levels are not different between the segregants that inherited BY and RM SNP 12_662,627 marker (*p* value of 0.178, the negative log of which is 0.75). (D) Each protein linkage with a *p* value less than 0.05 is plotted according to the negative log of the *p* value for linkage on the *x*-axis and the negative log of the *p* value for linkage after protein levels have been regressed on transcript levels on the *y*-axis. The horizontal line at 1.3 indicates the cutoff for significant linkage after normalization for transcript levels, and the three proteins that lose linkage after regression are indicated by arrows. Points close to the diagonal line, like those in the gray oval, are essentially unaffected by normalization for transcript levels. Two points were omitted to help with scale. The single point at coordinates (9.22, 0.75) whose calculation is described in panels A through C is noted.

This subset of 20 linkages comprise the most likely examples of loci that control the levels of proteins in the protein amino acid network primarily by controlling the underlying transcripts, but even here it is possible that transcription is not the main driver of protein levels. For example, a response to alterations in cellular physiology created by polymorphisms on chromosomes 3 and 13 may include both a transcriptional response of a specific set of genes and changes in the translation of the corresponding transcripts and/or stability of the corresponding proteins. Multilevel control (i.e. transcriptional and posttranscriptional) of the same genes is a well-described phenomenon in response to environmental changes and in development that assures the magnitude and rapidity of response and that reinforces cellular decisions [28],[29]. If the translational or protein stability changes have a larger effect on protein levels than the transcriptional alterations, one would still see shared genetic regulation and high correlation coefficients, but the protein network would not be driven primarily by transcription (Figure 1). To distinguish between these possibilities, we asked whether these 20 linkages for proteins in the amino acid network maintained linkage after normalizing for transcript levels [30]. For each of the proteins and the sites to which they are linked, such as ACS2 to chromosome 12, which is shown as an example (Figure 4A), protein levels were regressed on the corresponding transcript levels (Figure 4B), the residuals were tested for linkage to the original loci (Figure 4C), and residual linkage was plotted against the original linkage (Figure 4D). In the case of ACS2, it is clear that the locus on chromosome 12 is regulating ACS2 protein levels by regulating transcript levels, because the tight linkage between the locus and protein levels (*p* = 6.03×10^−10^, Figure 4A) becomes insignificant when the effect of the locus on transcript levels is taken into account (*p* = 0.178, Figure 4C). Two other linkages also behaved this way; thus a total of three out of the 20 protein linkages examined appear to reflect primarily transcriptional regulation of protein levels (three points below horizontal line at −log 0.05 in Figure 4D, in which all protein linkages have been plotted). Two of these three map to the transcription factor HAP1, which is inactivated in the laboratory parent by a Ty element insertion. These two genes, ACS2 and ERG6, are both regulated by HAP1 and both have upstream HAP1 binding sites that are among the most tightly HAP1-bound sites in the genome, thus suggesting a mechanism for transcript-mediated regulation of these two proteins [31]. Extending this test for transcript-mediated control of protein levels to all 179 protein linkages (*p* = 0.05) shows that the three cases mentioned above are the only instances in which control of protein levels can be attributed exclusively to control of the corresponding transcript (Figure 4D).

### Comparison of Cis-Regulation for Proteome and Transcriptome

**Figure 5:**
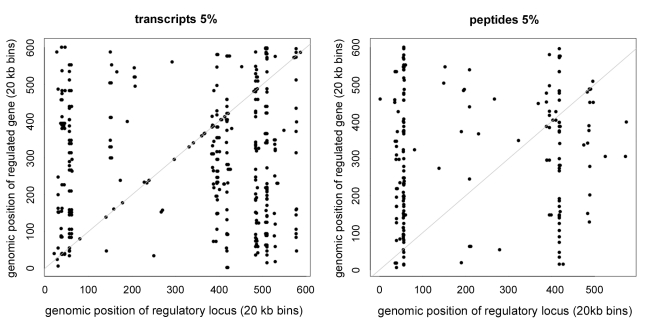
cis-linkage for the proteome versus the transcriptome. Each transcript linkage (*p*<0.05) is plotted on the left according to the location of the regulatory locus on the *x*-axis and the regulated gene on the *y*-axis, and the same is done for proteins on the right. Vertical strips indicate hot spots and points falling on the diagonal (gray line) indicate cis-linkage, i.e. the location of the genetic regulator is the same as that of the regulated gene.

Quantitative trait loci that affect transcript levels have been classified according to whether the regulated gene is linked (cis-regulation) or unlinked (trans-regulation) to the regulatory locus. For example a promoter mutation would be classified as cis-regulatory whereas a mutation in a transcription factor would likely appear as trans-regulatory. Consistent with numerous reports in both this collection of yeast strains and other populations [32]–[34], we find that at the transcript level, cis regulation of the 354 genes in this study is relatively common (Figure 5): at 1% FDR, we map regulators of 170 transcripts, 22 of which act in cis. In contrast, at this FDR we map regulators of 89 proteins, only three of which act in cis (Figure 5). *t* tests between each gene and a single cis marker to reduce the problem of multiple testing confirmed the wider prevalence of cis linkage in the transcriptome over the proteome: At a 1% significance, 50 transcripts show linkage to the nearest marker whereas only 13 proteins do. Normalizing for the 3.54 false positives expected suggests approximately 5-fold more cis linkage for transcripts than for proteins. As others have noted, we saw that cis regulators tended to have above average effect sizes and therefore should be easier to detect; for example, cis linkages at 1% significance explained on average 29% of variation in transcript levels whereas the corresponding trans linkages explained only 24%. Thus if variation in protein levels between individuals were caused by variation in the underlying transcripts, inaccurate measurements of either proteins or transcripts would lead to an overestimate of cis linkage, whereas we see the opposite. Cis linkage for transcript and protein abundance can be due to polymorphisms in the promoter region that alter transcription rates, or polymorphisms in the untranslated or coding sequences that alter transcript stability, translation rate, or protein stability. The virtual absence of cis linkage for proteins in the face of relatively common cis linkage for transcripts is consistent with our finding that variation in protein levels in this cross is largely independent of transcriptional variation. Furthermore, the rarity of cis linkage for proteins also suggests that cis-acting polymorphisms that lead to alterations in protein stability and/or translation rates are less common than those that alter transcription rates.

## Discussion

It is particularly noteworthy that, although ribosomal proteins and their corresponding transcripts are both tightly co-regulated, they are regulated entirely independently of one another; it is as if the loci regulating ribosomal transcripts are actively avoiding those that regulate the corresponding proteins, since permutation testing (with 10,000 permutations) demonstrates that there is less than a one in 10,000 chance that this level of “avoidance” could have happened by chance. This suggests that the loci that trigger the transcriptional response for ribosomal protein genes are acting through one pathway while the loci that trigger the corresponding translational (or protein stability) response are acting through another. The pathways that regulate transcription of ribosomal protein genes have been studied extensively. For example, nutrients activate the TOR and PKA pathways, which leads to phosphorylation of Sch9 and Sfp1. This increases the levels of Sch9 and causes Sfp1 to enter the nucleus, and this in turn triggers Fhl1- and Ifh1-dependent transcription of ribosomal protein and biogenesis genes [35],[36]. Thus the loci that regulate transcripts in the ribosomal community are likely perturbing intracellular physiology in a way that elicits a TOR and/or PKA signaling response.

**Figure 6:**
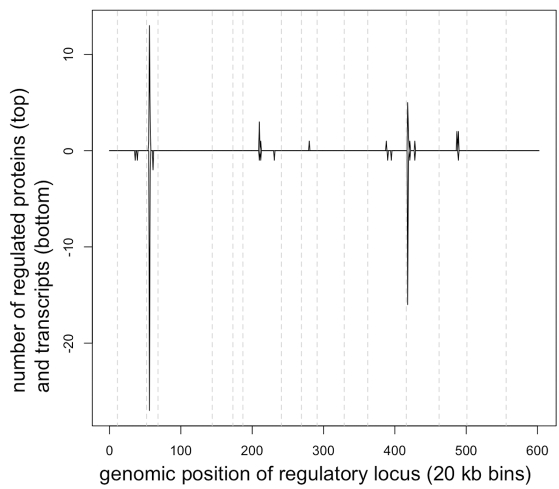
Similarity between genetic regulation of amino acid transcripts and ribosomal proteins. Loci that influence transcript and protein levels (*p*<0.05) are plotted as in Figure 2B–D, but regulators of proteins in the protein ribosomal community are plotted above the horizontal line while regulators of transcripts in the transcript amino acid community are plotted below the line.

So which pathway might regulate the translational (or protein stability) response that influences ribosomal protein levels? The loci that regulate the protein levels for genes in the protein ribosomal community (upper left in Figure 2) showed a striking resemblance to the loci that regulate transcripts in the transcript amino acid community (lower right in Figure 2; Figure 6), suggesting that these two networks are regulated by a common pathway. Furthermore, the major loci that regulate these genes on chromosomes 3 and 13 had diametrically opposing effects on the two groups; i.e. one allele of chromosome 3 caused essentially every amino acid transcript (65/67) to increase while also causing every ribosomal protein (36/36) to decrease, and the same was true for the locus on chromosome 13. Thus it appears that a single pathway is causing ribosomal proteins and amino acid transcripts to vary in opposition to each other. Our search for a pathway that regulates ribosomal protein translation therefore led us to consider pathways that affect transcription of genes involved in amino acid synthesis. The general amino acid control pathway (GAAC) responds to amino acid imbalances (sensed through levels of uncharged tRNAs) by activating Gcn2, which then phosphorylates the translation initiation factor eIF2A. This phosphorylation causes eIF2A to downregulate translation of a large number of genes while simultaneously promoting translation of the transcription factor GCN4, which in turn stimulates transcription of genes involved in amino acid synthesis [37]. Thus we suggest that loci on chromosomes 3 and 13 skew levels of uncharged tRNAs leading to a GAAC-dependent response that includes increasing transcription (via GCN4) of genes involved in amino acid biosynthesis and decreasing translation of ribosomal proteins. Likely candidate genes are LEU2 on chromosome 3, which is involved in leucine biosynthesis and is heterozygous in this cross and BUL2 on chromosome 13, which regulates amino acid import and is also heterozygous in this cross [38]. mRNAs encoding ribosomal proteins in higher eukaryotes have 5′ terminal oligopyrimidine tracts that are critical in regulating translation and thus protein levels [39]. The fact that yeast lack these so-called TOP mRNAs coupled with the observation that ribosomal protein transcripts in yeast are tightly regulated by the TOR signaling pathway [35],[36] has led to the widespread belief that yeast are different from other eukaryotes in that they regulate ribosomal protein levels primarily through transcription. Our observations suggest an important translational role for control of ribosomal protein levels in yeast, just like in vertebrates.

In summary, our results reveal striking differences in the effect of genetic variation on networks of proteins versus transcripts. Our results demonstrate that, in this genetically diverse population, levels of variation in transcripts are such that non-transcriptional mechanisms for controlling protein levels obscure the correlation between transcripts and proteins for most genes. This is not a consequence of selecting a subset of 354 genes whose transcripts were biased for low variance; on the contrary, every one of these transcripts was among the 522 with the highest variance, suggesting that even among highly variant transcripts, non-transcriptional variation is the predominant cause for variation in protein levels in this population. Finally, advances in proteome profiling technologies that enable high throughput profiling of low abundance proteins [40]–[42] combined with studies of other populations will help determine how general the predominance of non-transcriptional control is in controlling protein levels in genetically diverse populations, since our current analysis is limited to high abundance proteins. Given that protein abundance is more highly related to phenotype than transcript abundance, our findings underscore the fundamental role of non-transcriptional mechanisms in the creation of phenotypic diversity in genetically outbred populations.

## Materials and Methods

Raw data for both protein [14] and transcript [17],[19],[21] quantitation came from previously published work. Protein quantification in this manuscript is novel, i.e. the algorithm to extract protein quantitation from published mass spectrometric data sets is novel. Protein-protein and transcript-transcript correlation coefficients were calculated for each pair of genes. Segregant identities were then “scrambled” for one of the two measurements. For example, in calculating the correlation between levels of CDC19 protein and OLA1 protein, segregant identities would be shifted by 1 such that we calculated correlations on the basis of the level of CDC19 in segregant 2 with OLA1 levels in segregant 1, CDC19 levels in segregant 3 with OLA1 levels in segregant 2, and so forth. For each pair of proteins and for each pair of transcripts, 1,000 such permutations were performed. A 1% FPR was determined as higher than the 10th highest correlation coefficient calculated with the 1,000 permutations. Each pair of proteins and each pair of transcripts was thus assigned its own 1% FPR cutoff and this FPR was arbitrarily chosen as a cutoff for whether two proteins or transcripts are connected.

Communities were identified as previously described [23]. They were all derived from the same group of 354 genes for which both protein and transcript quantitation was available. The five closely related ribosomal protein communities consisted of five 35-member communities made up exclusively of 36 genes. Enrichment for functionally related genes within communities was calculated based on the fraction of members of a community with appropriate GO term assignments within the community as compared to the fraction of the same GO term assignments in the set of 354 genes.

### Heritability

Heritability is the degree to which variation in measurement is due to individual-to-individual variation as opposed to variation due to unintended fluctuation in experimental conditions. For each of 95 strains, there are between zero and four measurements for each protein level, 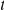 total measurements for each protein level across all segregants, *n*_1_ of which come from strain 1, *n*_2_ of which come from strain 2, and so forth. Heritability was calculated as follows:
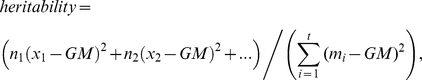
Where *x_i_* is the mean of the *i^th^* segregant, *m_i_* is the *i^th^* measurement, and *GM* is the “grand mean,” i.e. the mean of the entire population.

### Variance Explained

We use variance explained to quantify that proportion of the variation in phenotype (e.g. protein level) can be attributed to inheritance of a particular genetic marker. For example, imagine that the level of Aro1 protein is higher among segregants that inherited a SNP at base pair 10,000 on chromosome 3 from the RM parent than among segregants that inherited this SNP from the BY parent. *mean_RM_* is the mean level of Aro1 protein among segregants inheriting the RM SNP and *mean_BY_* is the mean level among segregants inheriting the BY SNP. *GM* is the “grand mean,” i.e. the mean level of Aro1 protein in all of the segregants. There are *k* total segregants with *i* segregants inheriting the RM SNP and *j* segregants inheriting the BY SNP. *m_RM_*__1_ is the Aro1 protein level in the first segregant inheriting the RM SNP, *m_RM_*__2_ is the level in the second segregant inheriting this SNP, and so forth.
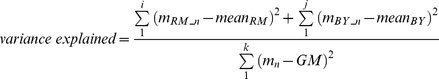
Data collection has been described previously [14]. The protein quantitation algorithm is described in detail below. Complete code is available from D.R. upon request. Data collection has been described previously [14]. The mapping algorithm was based on *t* tests with permutation testing to determine false discovery rates. Complete code is available from E.J.F. upon request. Communities were identified as described previously [23]. All protein measurements will be placed in the GEO online database upon publication (http://www.ncbi.nlm.nih.gov/geo/). Transcript measurements are available at this site.

## Supporting Information

Figure S1 Data of Lu et al. measuring protein and transcript levels for 346 genes in yeast were divided into groups within which transcript levels varied less than 3.5-fold and average correlation coefficients were calculated within each bin. The horizontal line at 0.31 shows the average cutoff for significance (*p*<0.01), as determined with 1,000 permutations.
(TIF)

Figure S2 Illustration of the difference between transcript-protein correlations between different genes in a single individual versus transcript-protein correlations between the same gene in different individuals.
(TIF)

Figure S3 Each of 354 genes is plotted according to the number of transcript-transcript connections (correlation coefficients with *p*<0.01) on the *x*-axis and protein-protein connections on the *y*-axis.
(TIF)

Figure S4 Zoom in view of isotopic strips. *X*-axis shows scan numbers and *y*-axis shows m/z.
(TIF)

Figure S5 Yellow pixels indicate the peptide peaks that are identified by MS2. On a typical run we identify 1,000 out of 5,000 to 6,000 peaks.
(TIF)

Table S1 Quantitation of 354 proteins.
(XLS)

Table S2 Community membership.
(DOC)

Table S3 Protein-transcript correlation coefficients.
(XLS)

Table S4 Genetic loci regulating protein and transcript levels.
(XLS)

Text S1 Protein quantitation algorithm (see also Figure S4 and S5).
(DOC)
