# Supplementary figures and images for: Genetic Variation Shapes Protein Networks Mainly through Non-transcriptional Mechanisms
Source: PLoS Biol. 2011 Sep 6;9(9):e1001144. doi: 10.1371/journal.pbio.1001144 (PMC3167781; doi:10.1371/journal.pbio.1001144)

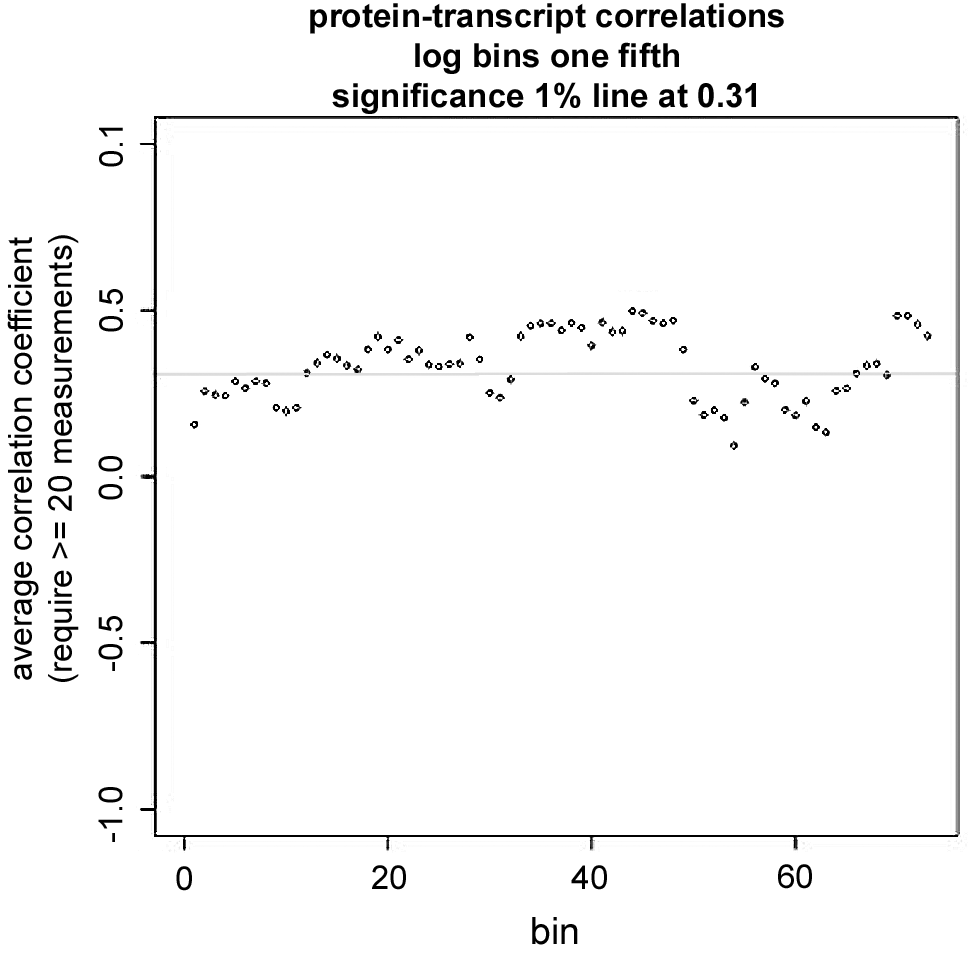

Supplement: Figure S1 — Data of Lu et al. measuring protein and transcript levels for 346 genes in yeast were divided into groups within which transcript levels varied less than 3.5-fold and average correlation coefficients were calculated within each bin. The horizontal line at 0.31 shows the average cutoff for significance (p<0.01), as determined with 1,000 permutations. (TIF) [file pbio.1001144.s001.tif]

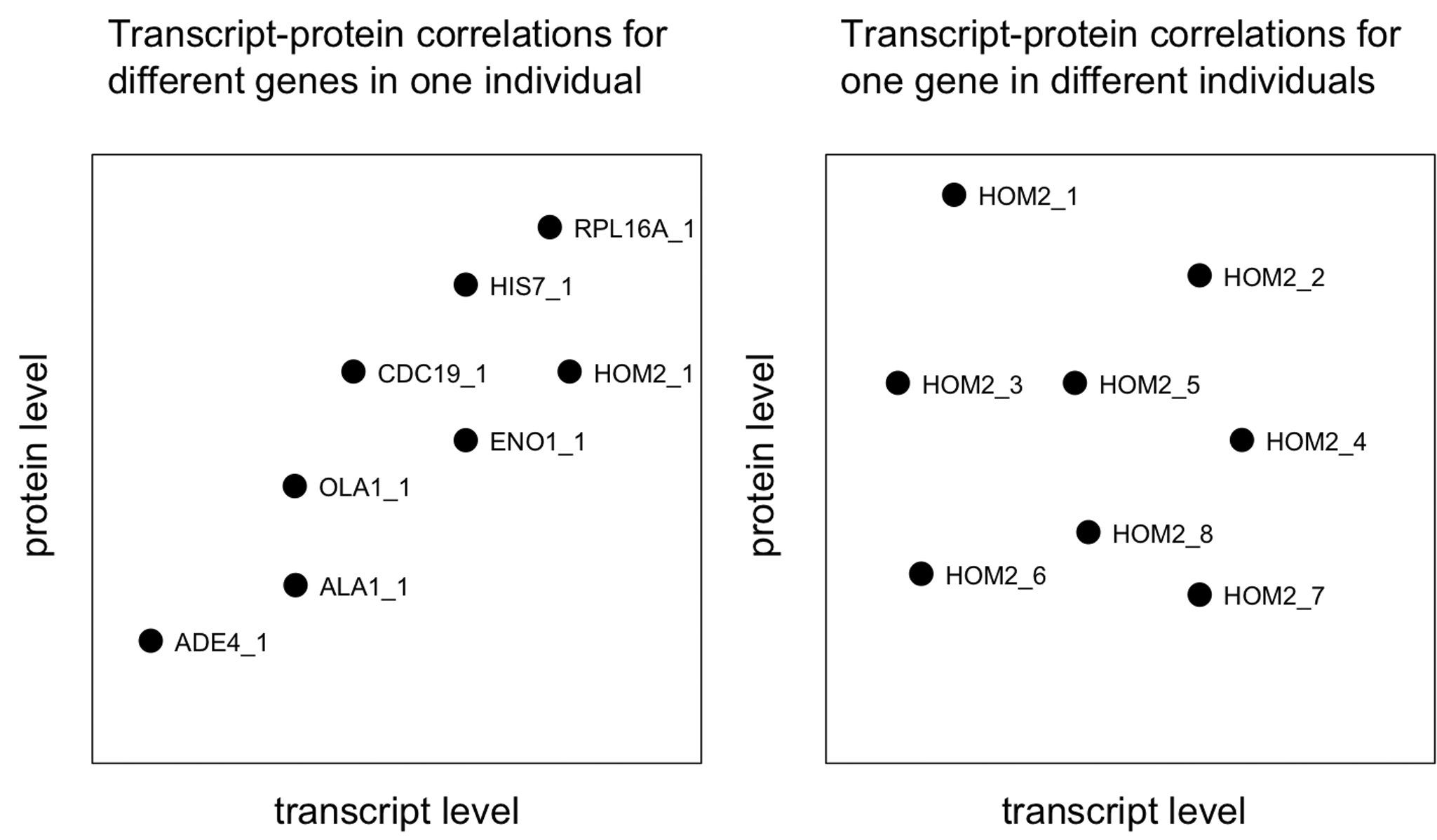

Supplement: Figure S2 — Illustration of the difference between transcript-protein correlations between different genes in a single individual versus transcript-protein correlations between the same gene in different individuals. (TIF) [file pbio.1001144.s002.tif]

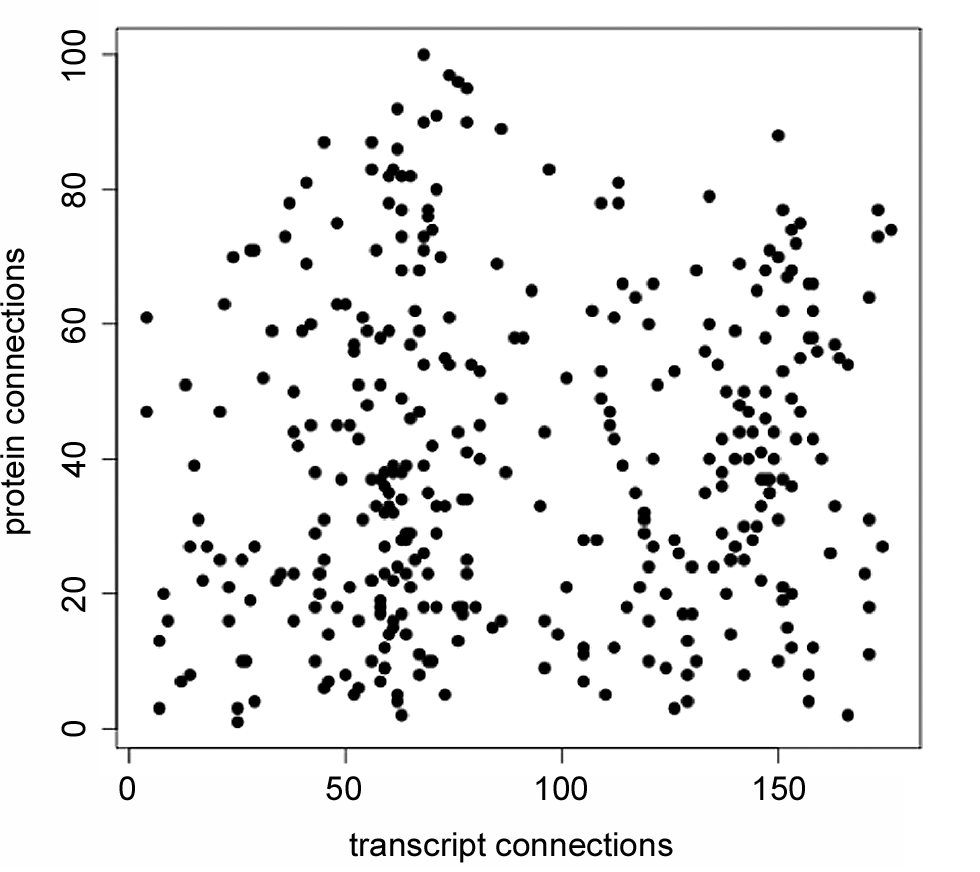

Supplement: Figure S3 — Each of 354 genes is plotted according to the number of transcript-transcript connections (correlation coefficients with p<0.01) on the x-axis and protein-protein connections on the y-axis. (TIF) [file pbio.1001144.s003.tif]

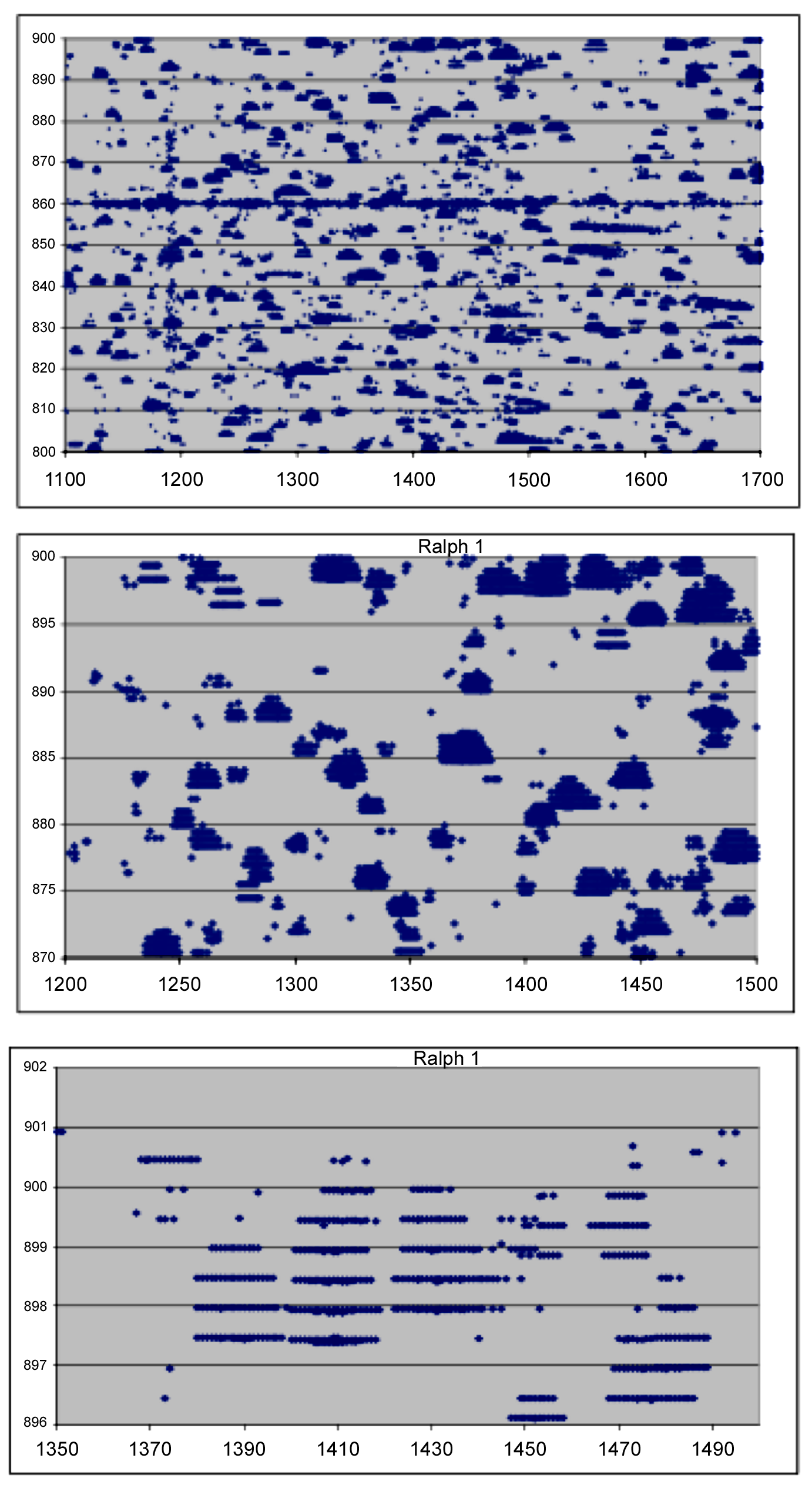

Supplement: Figure S4 — Zoom in view of isotopic strips. X-axis shows scan numbers and y-axis shows m/z. (TIF) [file pbio.1001144.s004.tif]

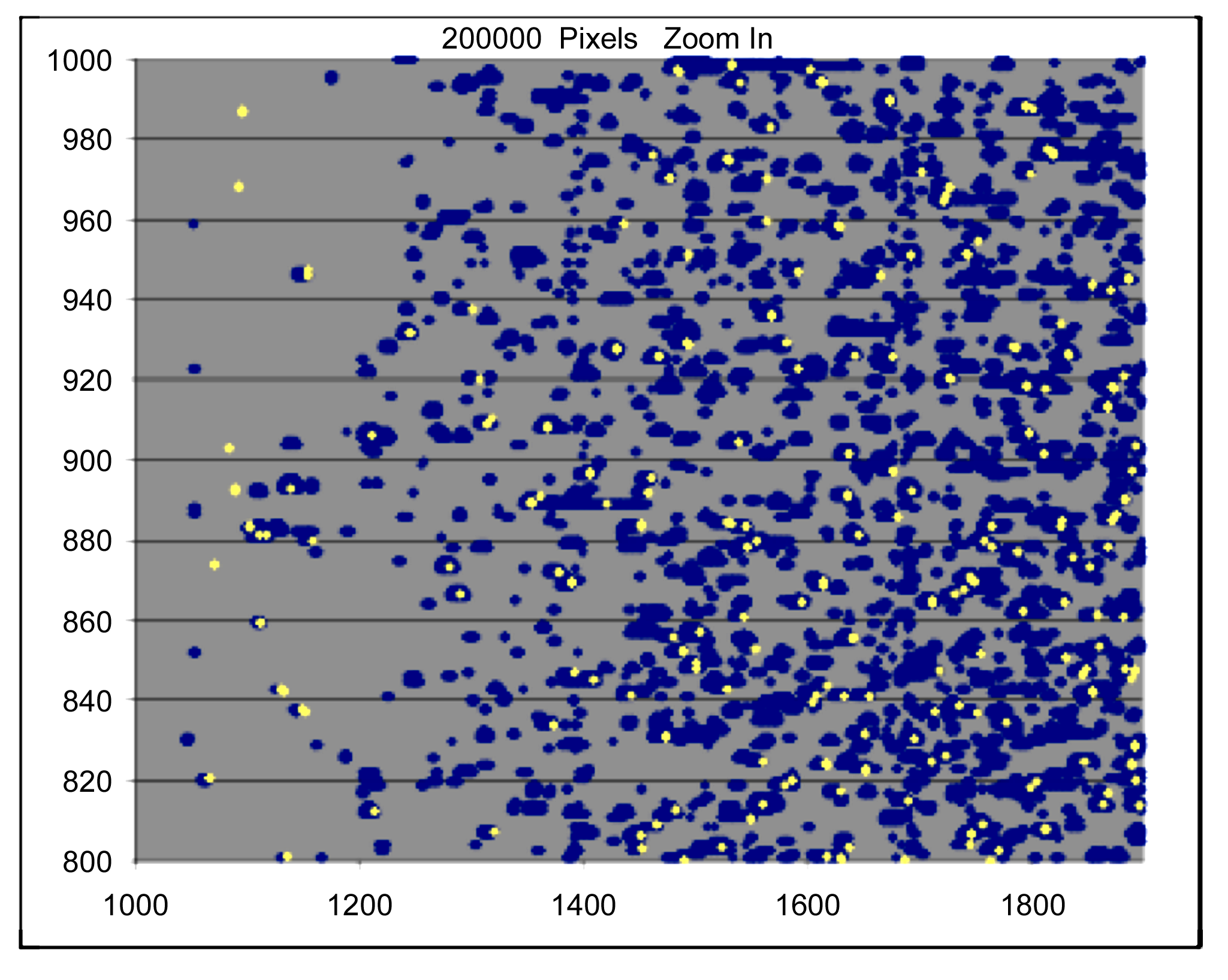

Supplement: Figure S5 — Yellow pixels indicate the peptide peaks that are identified by MS2. On a typical run we identify 1,000 out of 5,000 to 6,000 peaks. (TIF) [file pbio.1001144.s005.tif]
