# Supplementary material for: Genetic Variation Shapes Protein Networks Mainly through Non-transcriptional Mechanisms
Source: PLoS Biol. 2011 Sep 6;9(9):e1001144. doi: 10.1371/journal.pbio.1001144 (PMC3167781; doi:10.1371/journal.pbio.1001144)
Supplement: Table S2 — Community membership. (DOC) [file pbio.1001144.s007.doc]

Table S-1. Community membership.

| peptide_amino_acid | transcript_amino_acid | peptide_ribosome | transcript_ribosome |
| --- | --- | --- | --- |
| AAT2 | AAT2 | ALD6 | ADH1 |
| ACS2 | ADE3 | BGL2 | ADH2 |
| ACT1 | ADE4 | ENO2 | ADK1 |
| ADE3 | ADE5,7 | FAS1 | ADO1 |
| ADK1 | ADE6 | GFA1 | ALA1 |
| ALA1 | ADE8 | GNT1 | ALD6 |
| ALD5 | ALD5 | HSP60 | ARB1 |
| ARG1 | ARG1 | HXK1 | ARC1 |
| ARO2 | ARG5,6 | ILS1 | ARF1 |
| ARO3 | ARG8 | LYS12 | ASC1 |
| ARO4 | ARO1 | MRPL35 | CCT2 |
| ARO8 | ARO2 | NOP56 | CDC19 |
| ARP3 | ARO3 | OLA1 | CDC33 |
| ASN1 | ARO4 | OSH2 | CKA2 |
| ATP2 | ARO8 | PDI1 | CLU1 |
| BAT1 | ARP3 | RPL10 | CPR6 |
| BMH1 | ASN1 | RPL16A | CYS3 |
| BMH2 | BAT1 | RPL33B | CYS4 |
| CCT2 | CMD1 | RPL38 | DBP2 |
| CDC19 | DLD3 | RPL5 | DED1 |
| CPR3 | DPS1 | RPL9A | DED81 |
| CPR6 | ECM40 | RPP0 | DIS3 |
| DLD3 | GCV1 | RPP2A | ECM32 |
| DPM1 | GCV3 | RPS13 | EFB1 |
| EGD1 | GDH1 | RPS2 | EGD1 |
| ENO1 | GLN1 | RPS20 | EGD2 |
| ERG10 | GLT1 | RPS21B | FPR3 |
| ERG20 | GLY1 | RPS3 | FRS1 |
| ERG6 | HIS1 | RPS5 | FRS2 |
| FBA1 | HIS4 | RPS7A | GAS1 |
| FPR1 | HIS7 | SSA2 | GIS2 |
| GDH1 | HOM2 | TEF4 | GNT1 |
| GLY1 | IDP1 | THR4 | GRS1 |
| GUK1 | ILS1 | YDJ1 | GUA1 |
| GUS1 | ILV1 | YHR020W | GUK1 |
| HAS1 | ILV2 | YTA6 | GUS1 |
| HMO1 | ILV3 |  | HAS1 |
| HOM2 | ILV5 |  | HSP82 |
| HSP10 | KRS1 |  | HXK2 |
| IMD3 | LEU1 |  | HYP2 |
| KRS1 | LEU4 |  | IMD2 |
| LEU1 | LYS1 |  | IMD3 |
| LEU4 | LYS12 |  | IPP1 |
| LSP1 | LYS20 |  | KAP123 |
| LYS1 | LYS21 |  | KTR7 |
| LYS20 | MAE1 |  | NIP1 |
| LYS21 | MET17 |  | NOP1 |
| MET17 | MET6 |  | NOP56 |
| MMF1 | OAC1 |  | NPL3 |
| NOP1 | PDA1 |  | NSR1 |
| NSR1 | PGI1 |  | NTF2 |
| NTF2 | PRO2 |  | OLA1 |
| PDA1 | RHR2 |  | PDC1 |
| PEP4 | RNR4 |  | PFK1 |
| PFY1 | SER1 |  | POL32 |
| PGI1 | SHM1 |  | PUP3 |
| PGK1 | SHM2 |  | RNA1 |
| PMI40 | THR4 |  | RPA190 |
| POR1 | TRP2 |  | RPL10 |
| PRO2 | TRP5 |  | RPL13B |
| PST2 | TYS1 |  | RPL15A |
| PXA2 | VAS1 |  | RPL16A |
| RHR2 | YDR341C |  | RPL17A |
| RNR4 | YHR020W |  | RPL21A |
| ROT2 | YJL171C |  | RPL22A |
| RPL8A | YLR179C |  | RPL24A |
| RPS7B | YSA1 |  | RPL24B |
| RTN1 |  |  | RPL25 |
| SAM1 |  |  | RPL28 |
| SAM2 |  |  | RPL3 |
| SCP160 |  |  | RPL32 |
| SGT2 |  |  | RPL33B |
| SHM2 |  |  | RPL38 |
| SOD2 |  |  | RPL4A |
| SSE1 |  |  | RPL5 |
| STM1 |  |  | RPL6A |
| SUI2 |  |  | RPL6B |
| SYC1 |  |  | RPL8A |
| TIM9 |  |  | RPL8B |
| TPI1 |  |  | RPL9A |
| TRR1 |  |  | RPL9B |
| TRX2 |  |  | RPP0 |
| UNG1 |  |  | RPP1B |
| URA2 |  |  | RPP2A |
| WTM1 |  |  | RPP2B |
| YDL124W |  |  | RPS12 |
