# Supplementary material for: Genetic Variation Shapes Protein Networks Mainly through Non-transcriptional Mechanisms
Source: PLoS Biol. 2011 Sep 6;9(9):e1001144. doi: 10.1371/journal.pbio.1001144 (PMC3167781; doi:10.1371/journal.pbio.1001144)
Supplement: Text S1 — Protein quantitation algorithm (see also Figure S4 and S5). (DOC) [file pbio.1001144.s010.doc]

**Supplemental Online Material and Methods.**

**Protein quantitation algorithm**

Modern mass spectrometers, like the linear ion trap-Fourier transform ion cyclotron resonance (LTQ-FT) hybrid mass spectrometer in which our mass spectra were collected, have higher mass accuracy (low parts per million) and resolution (>100,000) than stand-alone ion traps such as the LTQ. This allows one to measure a peptides’ distribution of natural abundance of isotopes, which should allow for markedly better noise filtering as well as more accurate distinction of co-eluting peptides. We developed a suite of algorithms that made full use of these data, yielding data sets that with approximately ten-fold more MS1 features than our previous sets. These larger data sets, while more information-rich than the previous sets, are much more difficult to align because the alignment problem scales quadratically; thus a ten fold size increase translates into a hundred fold increase in computational cost, and this proved impractical. Thus we developed a completely new quantitation algorithm that is suitable for implementation on a standard computer under reasonable time constraints (a few days).

initial data extraction and notation

The raw LC-MS data files are converted to text format using Thermo Electron’s X-Calibur software. A Perl script is used to extract data points in the format $(h_{j},m_{j},I_{j})$, where $h_{j}$ is a scan number (used as a time stamp), $m_{j}$is the mass to charge ratio (m/z) and $I_{j}$is the ion current intensity (used for relative quantitation). Data are stored in a matrix, $P_{h,m}$, where h = 1, …, maxScan and m = 1, …, maxM/Z. The matrix entries are $P_{h_{j},m_{j}}=I_{j}$.

Signal Filtering

a) Initial Raw Filtering

We performed an initial file reduction by discarding the lower intensity signals in different mass to charge bins, as previously described [1]. We then implemented the previously described [1,2] “M-N rule”, which extracts only those pixels that are M times higher than the median for N headers in a row. Initially we set the desired number of data points to 200-300 thousand (as opposed to 32-64 thousand in our previous algorithms). Filtering according to the “M-N” rule allowed us to drastically reduce the size of our data files while still retaining most of the interesting features.

b) detection of isotopic “strips”

Since peptides elute over time during the LC phase of experiment and since peptides come with several isotopes, the LC-MS signature of a peptide should be detectable as several parallel horizontal strips. These strips should be very thin (the machine’s specification suggests that Y-axis reproducibility is +/- 0.02) and should last a few seconds up to a few minutes (peptide elution times). Figure S-4 confirms this.

A suite of algorithms was developed in order to extract these strips using a “top down” approach: First we identify the highest MS peak (i.e. $(h_{j},m_{j},I_{j})$ so that $I_{j}\geq I_{k}$for all k. Next we identify the pixels $(h_{j}\pm1,m_{j},I_{j})$, $(h_{j}\pm2,m_{j},I_{j})$, etc., thus extracting the horizontal strip containing the initial pixel. Once this strip is removed, we repeat the process.

c) Peptide Feature detection

Due to variation in peptides’ charge states (typically ranging from +1 to +5) and isotopic compositions, the LC-MS signature for a typical peptide consists of several horizontal strips separated by 1,1/2,1/3,1/4 or 1/5 Daltons. We developed a simple algorithm to reassemble these strips: Starting with largest strip, find a strip that has two properties: i) its time stamp is in a vicinity of the starting strip; and ii) its m/z signature is within the desired tolerance (i.e. 1,1/2,1/3,1/4 or 1/5). After detecting all the strips that pass this test we collect them into a peptide-signature and create $Pept=(X,Y,Z)$, where $X$ and $Y$ correspond to x and y coordinate (i.e. time stamp and m/z for the highest peak in the Bump) while $Z$ stands for the charge, which we infer from the distance between strips. Finally, we remove these strips from the file and repeat the procedure.

The final result of steps a, b, and c above is a sequence of data points called peptide features with notation $\{Pept_{i}\}_{i=1}^{N}$ where $Pept_{i}=(X_{i},Y_{i},Z_{i})$.

Alignment

At this point we typically have between 3000 and 7000 peptide features for each experiment and these peptide features must be aligned between experiments. Our alignment approach mimics our previously describe algorithms and the main innovations are related to the speed of convergence. In general we start with two data sets $Run_{1}=\{X_{i}^{1},Y_{i}^{1},Z_{i}^{1}\}_{i=1}^{N_{1}}$ and $Run_{2}=\{X_{j}^{2},Y_{j}^{2},Z_{j}^{2}\}_{j=1}^{N_{2}}$, for which we define the overlap function

$W(Run_{1},Run_{2})=\sum_{j=1}^{N_{1}} \min_{i=1,N_{2}} \phi(|X_{j}^{1}-X_{i}^{2}|,|Y_{j}^{1}-Y_{i}^{2}|)$,

where the weight function $\phi(x,y)=\frac{1}{(1+x^{2})(1+(100y)^{2})}$ is designed to penalize the misalignments. The y axis discrepancy is penalized more because m/z measurements are more accurately reproduced between experiments than are elution times ($X_{j}^{1}$ and $X_{i}^{2}$). One is, of course, free to choose different types of weight function suitable for a particular set of data points. (In particular one could relax the penalty for x-misalignments.) The task of alignment reduces to finding the time transform function F(t) such that overlap $W(Run_{1},Run_{2}^{F})$ is maximized. The $Run_{2}^{F}$stands for time shifted version of the original $Run_{2}$ data, i.e. $Run_{2}^{F}=\{F(X_{j}^{2}),Y_{j}^{2},Z_{j}^{2}\}_{j=1}^{N_{2}}$.

Finding such a function is an algorithmically intractable task, and in order to proceed we parameterize the problem. The curve F(t) is approximated by a linear spline function of the form:

$$F_{d}(t)=\sum_{k=1}^{d} (m{}_{k}t+n_{k})1_{t\in I_{k}}$$

where $I_{k}=[a_{k},a{}_{k+1}]$are disjoint intervals covering the time domain. The parameters $m_{k},n{}_{k}$ are chosen so that F(t) is a continuous function (i.e. $m{}_{k}a_{k}+n_{k}=m{}_{k+1}a_{k}+n_{k+1}$). This simple form now allows us to easily model the key requirement $1-\delta\leq F'(t)\leq1+\delta$ by requesting that . (Because we are familiar with the mechanism that causes the time shift, it is safe to assume that the F(t) is a smooth function with slope deviating very slightly from 1, and furthermore we have confirmed this assumption experimentally.) Consequently, the function of interest is parameterized by d+1 parameters ($n{}_{1},m_{1},...,m_{d})$, and our problem reduces to maximizing the function

$G(n{}_{1},m_{1},...,m_{d})=W(Run_{1},Run_{2}^{F_{d}})$ .

Although simplified the above optimization problem is still difficult. Our previously published results used d = 6, which allowed us to capture the curvature very well but it also made the optimization problem much more complicated. The main difficulty accompanying the new suite of algorithms is related to the ten-fold increase in data size (i.e. the number of peptides per run is 10 fold higher). The computational cost related to the target function W, scales quadratically (i.e. cost is $N_{1}N_{2}$) but it is possible to design an algorithm that decreases the computational cost to a more manageable $Const\cdot N_{1}Ln(N_{2})$. In our case the computational cost related to the evaluation of the weight function W, is *circa* 40 fold higher then the analogous cost for the study we reported in 2007 [1]. Since our previously published algorithm, when applied to this same set of LC-MS runs, required several hundred hours of computing time, this 40 fold increase clearly required a more efficient set of optimization tools. We did this in three steps:

Alignment step 1: Alignment of total ion current. Given the LC-MS run $P=\{h_{j},m_{j},I_{j}\}_{j=1,N}$we generate a function $C_{P}(k)=\sum_{j=1}^{N_{P}} I_{j}1_{h_{j}=k}$ (for a given header k we add all the intensity from pixels with header number $h_{j}=k$), which we approximate with a smooth function (we opted for cubic splines but polynomial or rational approximation could be used as well). This smoothed version$\tilde{C}_{P}(x)$ should be relatively simple with few parameters (5-6 parameters). Next, for two runs (say P and Q ) we find a time transformation Polynomial function g(t) in order to minimize . We used an accelerated random search (ARS) [3] optimization tool in order to find the appropriate g(t). The computational cost for this first step is very modest since the function $C_{P}(k)$needs to be computed only once.

Alignment step 2: Using the polynomial function g(t) as an initial approximation of the time transformation function F(t), we transform $Run_{2}:=\{g(X_{j}^{2}),Y_{j}^{2},Z_{j}^{2}\}_{j=1}^{N_{2}}$, and proceed with previously described optimization of $W(Run_{1},Run_{2}^{F_{d}})$. We use the ARS algorithm with the main advantage here that the initial step, using very little CPU time, provides a good “starting” point, which tends to speed up the overall optimization by a factor of 5-10. Additional speeding up comes from the spline dimension reduction. Namely, instead of using a 6-spline as we did in our previous algorithm, which yields a very difficult and time-consuming 7-dimensional optimization problem, here we use only a 3-Spline. This step provides another reduction of computational cost, typically by an order of magnitude, but it comes with a price; and for this we need the third step.

Alignment step 3: The first two steps are able to position the peptides from two LC-MS runs relatively close to each other. However, since we use only a 3-spline approximation we cannot capture all of the curvature and in many cases related peptides will not overlap. The final step exploits the extra precision provided by modern FTMS machines: For a given peptide from $Run_{1}$we look +/- 100 time units and find a peptide from $Run_{2}$ that has a) the same charge and b) m/z is within 0.02 Daltons tolerance. We experimentally confirmed that one should not significantly enlarge the +/- 100 time unit search interval.

generation of a peptide manifold

The final product of filtering and aligning is a pair-wise connection matrix M, where $M(Pept_{m,k},Pept_{i,j})=1$ indicates that m’th peptide from k’th run (i.e. $Pept_{m,k}$) corresponds to i’th peptide from j’th run (i.e. $Pept_{i,j}$). As previously described [1], we used graph theory to extract peptide arrays $Pa=V_{m_{1},1}\to V_{m_{1},2}\to...\to V_{m_{1},K}$, i.e. a sequence of numbers each corresponding to relative quantity of this particular peptide within each of the runs. In other words, for peptide array $m_{1}$, $V_{m_{1},1}$is the relative quantity in $Run_{1}$, while $V_{m_{1},2}$ is the relative quantity in $Run_{2}$ etc. In total, we extracted *circa* 9000 peptide arrays. At this point, we normalized our data to total ion current for each run.

Assignment of peptide sequences

Peptide sequences were identified by tandem mass spectrometry in the LTQ on the front end of the instrument, and these identifications were assigned to peptide peaks in the MS1 spectra after alignment. A typical run consists of 4000-6000 peptide peaks and we on average manage to identify 1000 of these peaks (Fig. S-5). Although, we identify only about 20% of peptides in each run, the alignment modulus allows us to bootstrap this figure to 70%; namely, in order to identify a peptide array we do not need to sequence that peptide in every run.

# converting peptide quantitation to protein quantitation

Applied to our data, the above algorithms produce *circa* 9000 peptide arrays, and circa 6000 of these have successful sequencing attached to them. In order to infer protein quantitation from peptide quantitation, we developed an algorithm to choose a single peptide to represent each protein. We have experimentally observed that a typical protein is represented by 1-10 different peptide sequences, and each of these peptide sequences is typically represented by 3-10 different peptide arrays. The latter observation reflects different charge states, single peptide peaks being unintentionally split into 2 and algorithmic errors such as incorrect alignments, feature extractions that fuse peptides that co-elute and have similar masses, and filtering algorithms that can filter out MS1 signals of low abundance peptides that can nonetheless be identified in the highly sensitive LTQ used for MS2 measurements. Consequently a median number of peptide sequences per protein is 9-10, while the range is much larger 3-100 peptide sequences per protein, and it is not obvious how to choose the peptide array that most accurately reflects the original protein’s abundance. The key feature that allowed us to solve this problem comes from the fact that we did not use any of the peptide sequencing information during our peptide array assembling. These sequences are identified by fragmentation in the LTQ and were performed completely independently from the quantification procedure, both algorithmically as well as physically.

Therefore we can use the MSMS peptide sequencing as an independent criteria to weed out most of the misalignment as well as to measure the quality of the peptide arrays:

For a typical peptide array

$$Pa=V_{m_{1},1}\to V_{m_{1},2}\to...\to V_{m_{1},K}$$

we have an associated peptide-sequencing array

$PaS=S_{m_{1},1}\to S_{m_{1},2}\to...\to S_{m_{1},K}$,

Where as before $V_{m_{1},j}$ stands for a total ion current for $m_{1}$’th bump in j’th run while $S_{m_{1},j}$ stands for the peptide sequence of the same bump. If a particular peptide peak is not sequenced, then $S_{m_{1},j}$ is blank. For a given peptide-sequencing array we define three quantities

MT = total number of non-blank sequencings $S_{m_{1},j}$,

ML = the largest number of repeating sequencing among MT detected sequences

MR = ML/MT .

For example, IPVLEQELVR, 77,77,1 indicates that for this particular peptide array there are 77 non-blank sequencings and all of them are identified as IPVLEQELVR. This would be an example of confirmed alignment. On the other hand, AQRPITGASLDLIK , 38, 22, 0.57 indicates that for this particular peptide array there are 38 non blank sequencings and that only 22 of them were identified as AQRPITGASLDLIK . Therefore there are 16 instances where the ostensibly aligned peaks were identified as different peptides. This peptide array would be discarded due incorrect alignment or sequencing.

The majority of peptide sequences have a quality ratio MR<1, which reflects the difficulty of getting all steps perfectly right (i.e. perfect alignment, sequencing and filtering). For this report, we kept only the sequences with ratio MR>4/5 . Our peptide selection procedure occurs in three steps:

Peptide selection step 1: Of 6000 peptide arrays that have sequences, we pick only the consistent ones, the ones for which the ratio MR > 0.8, as described above. This stringent test eliminates more than 50% of our data.

Peptide selection step 2: For a given peptide sequence we pick the best peptide array.

2a) For a given peptide sequence, sort the arrays based on MT and pick the top 50% (i.e. the arrays with largest number of sequencings);

2b) the remaining arrays are sorted based on MR and we again pick the top 50% (i.e. the arrays with the best ratio);

2c) finally, among remaining arrays we pick the one with highest total ion current.

Peptide selection step 3: Step 2 produces a list of quantified and sequenced peptides, each represented by its “best” peptide array. In order to choose the best peptide for each protein, we do the following:

3-a) Choose only those peptides that uniquely determine a protein and construct a list consisting of these proteins. Thus, for each protein on this list we will have a collection of peptides and their peptide arrays.

3-b) For a given protein sort the corresponding peptide arrays based on MT and pick the top 50% (i.e. the peptides with largest number of sequencings);

3-c) among the remaining list of peptides, sort based on MR and pick the top 50%;

3-d) and finally, among the remaining peptides pick the one with the largest total ion current and use this peptide as the representative for the given protein.

1. Foss EJ, Radulovic D, Shaffer SA, Ruderfer DM, Bedalov A, et al. (2007) Genetic basis of proteome variation in yeast. Nat Genet 39: 1369-1375.

2. Radulovic D, Jelveh S, Ryu S, Hamilton TG, Foss E, et al. (2004) Informatics platform for global proteomic profiling and biomarker discovery using liquid chromatography-tandem mass spectrometry. Mol Cell Proteomics 3: 984-997.

3. Appel MJ, Labarre R, Radulovic D (2004) On accelerated random search. Siam Journal on Optimization 14: 708-731.
